# Supplementary figures and images for: Pathway-specific effects of ADSL deficiency on neurodevelopment
Source: eLife. 2022 Feb 8;11:e70518. doi: 10.7554/eLife.70518 (PMC8871376; doi:10.7554/eLife.70518)

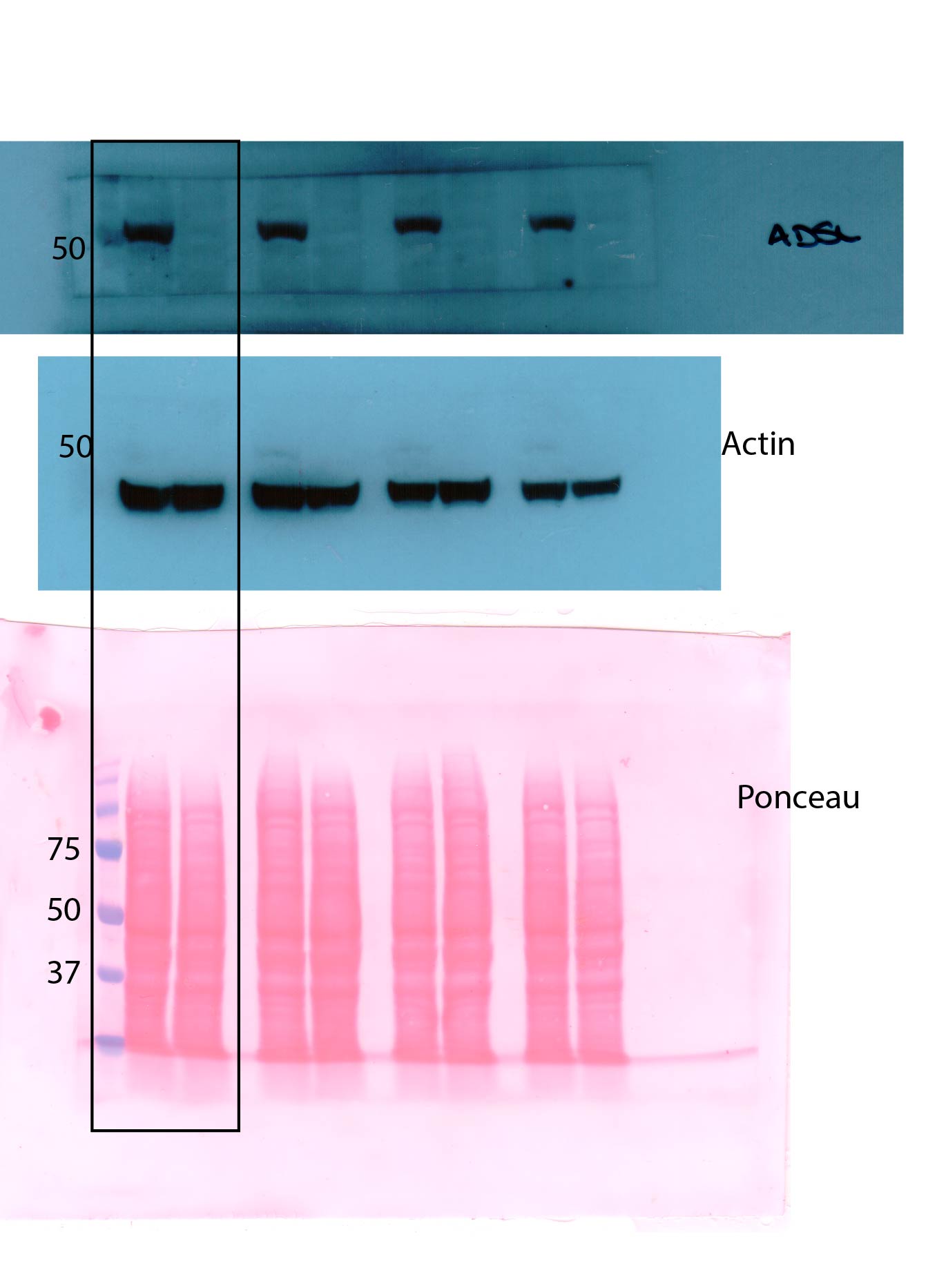

Supplement: Figure 1—source data 2. — jpg file. [file elife-70518-fig1-data2.zip › Fig1-source data 2.jpg]

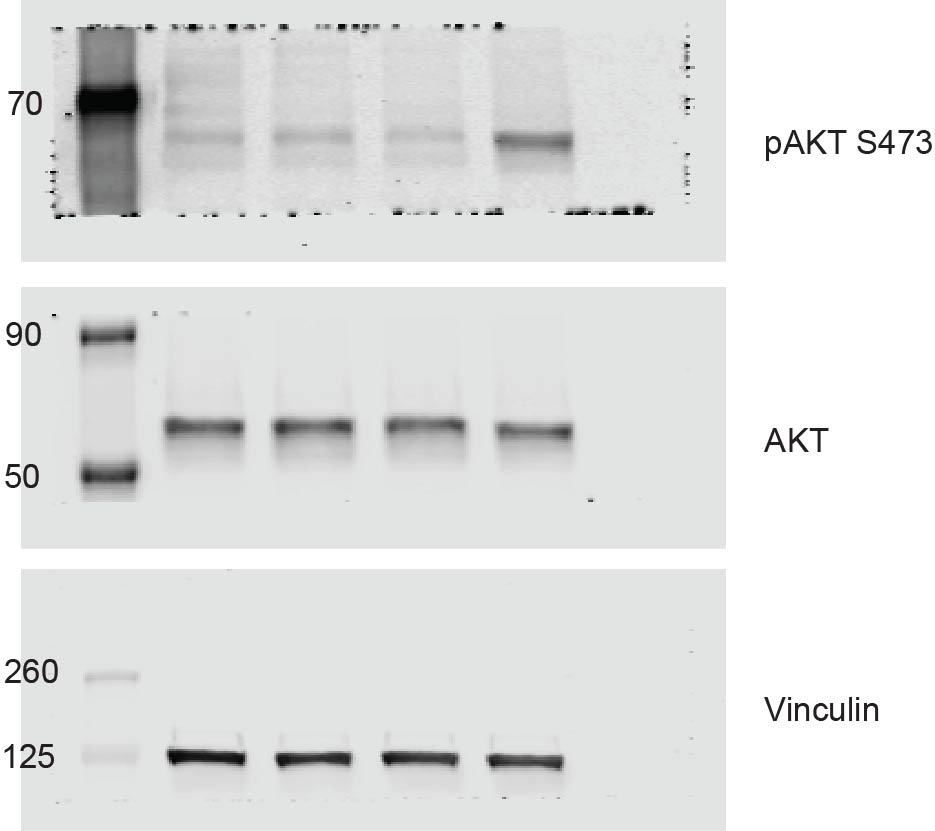

Supplement: Figure 4—figure supplement 1—source data 1. [file elife-70518-fig4-figsupp1-data1.zip › Fig4source data2.jpg]
